# Equine-Origin Immunoglobulin Fragments Protect Nonhuman Primates from Ebola Virus Disease

**DOI:** 10.1128/JVI.01548-18

**Published:** 2019-02-19

**Authors:** Hualei Wang, Gary Wong, Wenjun Zhu, Shihua He, Yongkun Zhao, Feihu Yan, Md Niaz Rahim, Yuhai Bi, Zirui Zhang, Keding Cheng, Hongli Jin, Zengguo Cao, Xuexing Zheng, Weiwei Gai, Jieying Bai, Weijin Chen, Yong Zou, Yuwei Gao, George F. Gao, Songtao Yang, Xianzhu Xia, Xiangguo Qiu

**Affiliations:** a Institute of Military Veterinary, Academy of Military Medical Sciences, Changchun, China; b National Microbiology Laboratory, Public Health Agency of Canada, Winnipeg, Manitoba, Canada; c Institut Pasteur of Shanghai, Chinese Academy of Sciences, Shanghai, China; d Département de Microbiologie-Infectiologie et d’Immunologie, Université Laval, Québec, Quebec, Canada; e Department of Medical Microbiology, University of Manitoba, Winnipeg, Manitoba, Canada; f CAS Key Laboratory of Pathogenic Microbiology and Immunology, Institute of Microbiology, Chinese Academy of Sciences, Beijing, China; g Jiangsu Co-innovation Center for Prevention and Control of Important Animal Infectious Disease and Zoonoses, Yangzhou University, Yangzhou, China; h Changchun Institute of Biological Products Co. Ltd., Changchun, China; St. Jude Children's Research Hospital

**Keywords:** Ebola virus, equine, immunoglobulin fragments, immunotherapy, nonhuman primates

## Abstract

Ebola virus (EBOV) infections result in aggressive hemorrhagic fever in humans, with fatality rates reaching 90% and with no licensed specific therapeutics to treat ill patients. Advances over the past 5 years have firmly established monoclonal antibody (MAb)-based products as the most promising therapeutics for treating EBOV infections, but production is costly and quantities are limited; therefore, MAbs are not the best candidates for mass use in the case of an epidemic. To address this need, we generated EBOV-specific polyclonal F(ab′)_2_ fragments from horses hyperimmunized with an EBOV vaccine. The F(ab′)_2_ was found to potently neutralize West African and Central African EBOV *in vitro*. Treatment of nonhuman primates (NHPs) with seven doses of 100 mg/kg F(ab′)_2_ beginning 3 or 5 days postinfection (dpi) resulted in a 100% survival rate. Notably, NHPs for which treatment was initiated at 5 dpi were already highly viremic, with observable signs of EBOV disease, which demonstrated that F(ab′)_2_ was still effective as a therapeutic agent even in symptomatic subjects. These results show that F(ab′)_2_ should be advanced for clinical testing in preparation for future EBOV outbreaks and epidemics.

**IMPORTANCE** EBOV is one of the deadliest viruses to humans. It has been over 40 years since EBOV was first reported, but no cure is available. Research breakthroughs over the past 5 years have shown that MAbs constitute an effective therapy for EBOV infections. However, MAbs are expensive and difficult to produce in large amounts and therefore may only play a limited role during an epidemic. A cheaper alternative is required, especially since EBOV is endemic in several third world countries with limited medical resources. Here, we used a standard protocol to produce large amounts of antiserum F(ab′)_2_ fragments from horses vaccinated with an EBOV vaccine, and we tested the protectiveness in monkeys. We showed that F(ab′)_2_ was effective in 100% of monkeys even after the animals were visibly ill with EBOV disease. Thus, F(ab′)_2_ could be a very good option for large-scale treatments of patients and should be advanced to clinical testing.

## Abstract (precis)

EBOV is one of the deadliest viruses to humans. It has been over 40 years since EBOV was first reported, but no cure is available. Research breakthroughs over the past 5 years have shown that MAbs constitute an effective therapy for EBOV infections. However, MAbs are expensive and difficult to produce in large amounts and therefore may only play a limited role during an epidemic. A cheaper alternative is required, especially since EBOV is endemic in several third world countries with limited medical resources. Here, we used a standard protocol to produce large amounts of antiserum F(ab′)_2_ fragments from horses vaccinated with an EBOV vaccine, and we tested the protectiveness in monkeys. We showed that F(ab′)_2_ was effective in 100% of monkeys even after the animals were visibly ill with EBOV disease. Thus, F(ab′)_2_ could be a very good option for large-scale treatments of patients and should be advanced to clinical testing.

## INTRODUCTION

First reported in 1976, Ebola virus (EBOV) infections in humans result in case fatality rates of up to 90%, with no approved medical countermeasures (1). Humans infected with EBOV initially present nonspecific, flu-like symptoms, including a sudden onset of fever, headaches, muscle and joint pain, and general fatigue and malaise (2), before progressing to gastrointestinal, respiratory, vascular, and neurological disease (1). A maculopapular rash accompanied by hemorrhagic symptoms may be observed in a proportion of patients (1). Terminal EBOV disease (EVD) is characterized by severe metabolic imbalances, anuria, convulsions, and hypovolemic shock, with death typically due to multiorgan failure and capillary extravasation from vascular permeability and diffuse coagulopathy (2). Recovery in survivors is slow, and neurological symptoms have been reported (3).

Historically, EVD outbreaks tend to be sporadic, unpredictable, and localized to isolated regions of sub-Saharan Africa, with fatalities numbering in the hundreds at most (4). However, an EVD epidemic spread throughout Guinea, Sierra Leone, and Liberia in 2014 to 2016, killing over 11,000 people (5). Two years later, back-to-back EVD outbreaks in the Equateur and North Kivu provinces in the western and eastern regions, respectively, of the Democratic Republic of the Congo has put EBOV back in the spotlight. In particular, the outbreak in North Kivu is proving to be very challenging to control, due to the volatile security situation (6).

EBOV-specific antibody levels are known to correlate statistically with survival from EVD (7). Monoclonal antibody (MAb)-based candidates, such as ZMAb (8, 9), ZMapp (10), and MIL-77 (11), are promising treatments (12–14), due to successes in the treatment and reversion of advanced EVD in humans and nonhuman primates (NHPs). In addition, ZMapp appears to have a beneficial effect on survival rates in clinical trials (15). However, MAb-based products can be expensive and time-consuming to manufacture in large quantities, thus restricting their usefulness in areas with limited resources. Polyclonal antisera containing EBOV-specific human IgG produced from transchromosomic cattle have been shown to be efficacious in NHPs when given at 3 days postinfection (dpi) (16), but the cows are transgenic and may not be widely available to researchers in developing countries if they wish to produce their own antibody-based products. An alternate option is passive immunotherapy with polyclonal immunoglobulin F(ab′)_2_ fragments produced from hyperimmunized horses. Advantages include high yields of antisera (120 to 250 g per horse [17]) in relatively short times, cost efficiency, low risk of contamination by adventitious agents, and standardized production procedures. F(ab′)_2_ fragments are still commonly used in African, Asian, and Latin American countries to treat envenomation via animal bites and/or stings and rabies (18) and Clostridium tetani (19) infections. Additionally, we previously showed that F(ab′)_2_ derived from horses hyperimmunized with a virus-like particle EBOV vaccine completely protected mice and guinea pigs *in vivo* when treatment was initiated 24 h after challenge (17). Our aim in this study was to characterize the protective efficacy of equine F(ab′)_2_ in NHPs.

## RESULTS

### *In vitro* neutralization properties of F(ab′)_2_ against EBOV.

**FIG 1:**
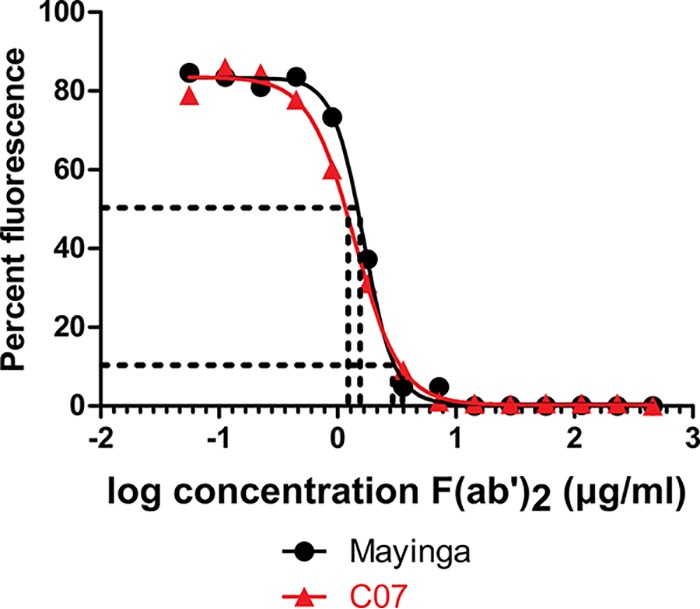
*In vitro* neutralizing activities of equine F(ab′)_2_ against EBOV-Mayinga-eGFP and Makona-C07-eGFP in VeroE6 cells. Neutralizing activities of F(ab′)_2_ against EBOV-Mayinga-eGFP or EBOV-C07-eGFP were compared over different F(ab′)_2_ concentrations (*x* axis). Fluorescence (*y* axis) from infected VeroE6 cells at 3 dpi is shown as a percentage of the fluorescence observed with the PBS control (set at 100%). Dashed lines indicate 50% or 90% inhibition of fluorescence and the associated F(ab′)_2_ concentrations.

We first tested the *in vitro* neutralizing activities of our current F(ab′)_2_ batch (which had been stored at 4°C for 33 months) against a recombinant, live, Central African (EBOV-Mayinga) (20) or West African (EBOV-Makona-C07) (21) EBOV strain expressing enhanced green fluorescent protein (eGFP). F(ab′)_2_ was found to be potently neutralizing against both tested viruses, with 50% effective concentration (EC_50_) values of 1.7 and 1.4 μg/ml against EBOV-Mayinga-eGFP and EBOV-Makona-C07-eGFP, respectively (Fig. 1). The 90% effective concentration (EC_90_) values were 3.2 and 3.7 μg/ml against EBOV-Mayinga-eGFP and EBOV-Makona-C07-eGFP, respectively (Fig. 1).

### Efficacy of F(ab′)_2_ at 3 dpi against EBOV in NHPs.

**FIG 2:**
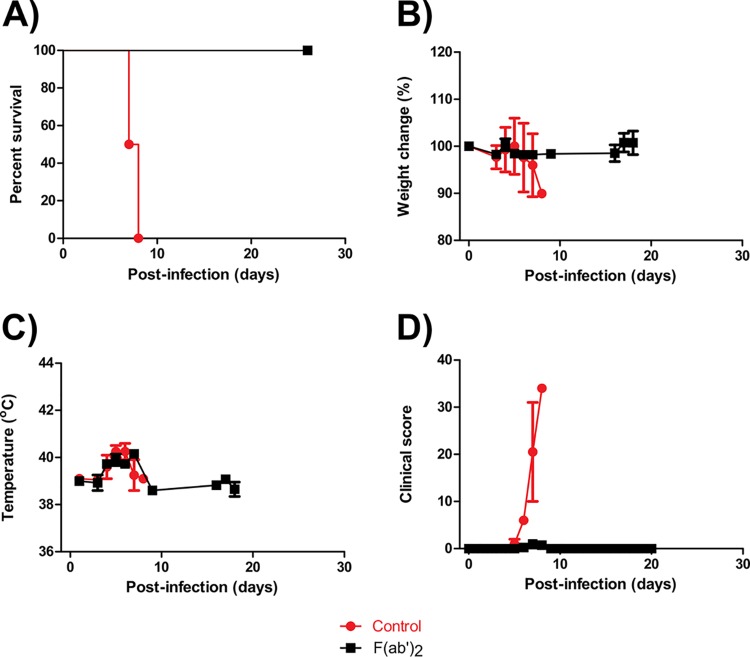
Survival rates and clinical findings for NHPs after EBOV challenge at 3 dpi. NHPs were given equine F(ab′)_2_ starting at 3 dpi. (A) Survival rates. (B) Percent weight changes. (C) Body temperatures. (D) Clinical scores.

**FIG 3:**
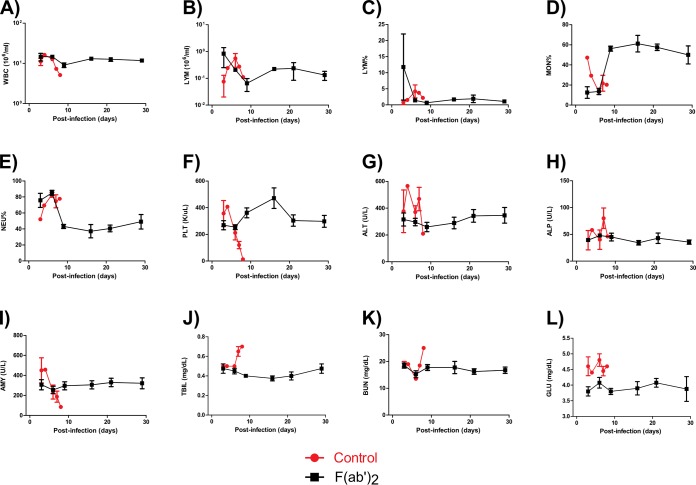
Hematology and serum biochemistry findings for NHPs after EBOV challenge at 3 dpi. NHPs were given equine F(ab′)_2_ starting at 3 dpi. (A) WBC counts. (B) LYM counts. (C) LYM percentages. (D) MON percentages. (E) NEU percentages. (F) PLT counts. (G) ALT levels. (H) ALP levels. (I) AMY levels. (J) TBIL levels. (K) BUN levels. (L) GLU levels.

Administration of F(ab′)_2_ resulted in 100% protection (Fig. 2A), and the F(ab′)_2_-treated NHPs did not lose substantial amounts of body weight during the experiment (Fig. 2B). Fever was observed at 4 to 7 dpi in all animals, but temperatures returned to baseline by 8 dpi (Fig. 2C), and F(ab′)_2_-treated NHPs showed virtually no observable signs of disease throughout the course of the experiment (Fig. 2D). In contrast, control animals died at 7 or 8 dpi with clinical scores of over 30 and symptoms consistent with EVD. Complete blood count results showed transient decreases in white blood cell (WBC) counts for 2 of 4 F(ab′)_2_-treated NHPs (Fig. 3A) but no substantial decreases in lymphocyte (LYM) counts or LYM percentages (Fig. 3B and C). Increases in monocyte (MON) percentages and decreases in neutrophil (NEU) percentages were observed for all F(ab′)_2_-treated NHPs (Fig. 3D and E). Changes in platelet (PLT) counts were not observed for any F(ab′)_2_-treated NHPs (Fig. 3F). In contrast, control animals showed decreases in WBC counts, MON percentages, and PLT counts, as well as increased NEU percentages, during the course of the experiment.

**FIG 4:**
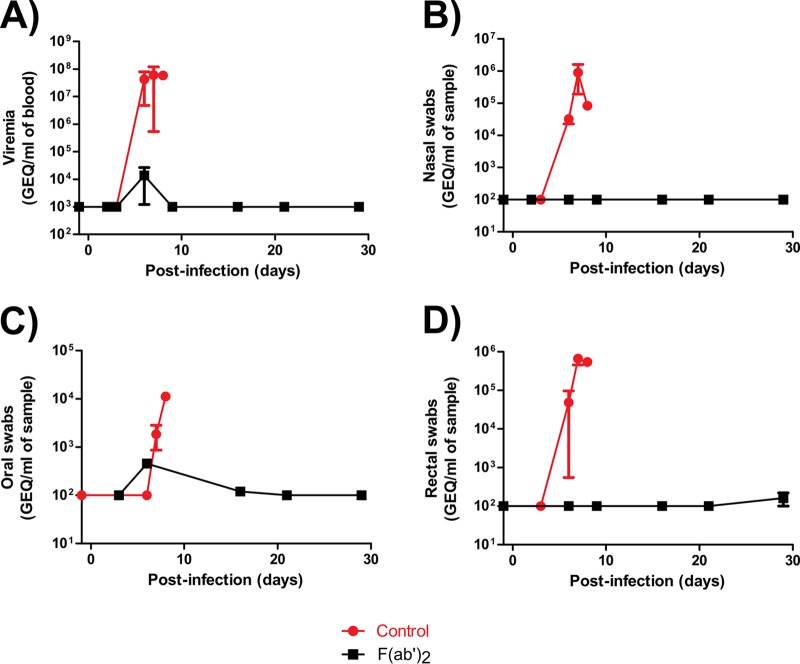
Viremia and shedding of NHPs after EBOV challenge at 3 dpi. NHPs were given equine F(ab′)_2_ starting at 3 dpi. (A) Blood. (B) Nasal swabs. (C) Oral swabs. (D) Rectal swabs.

**FIG 5:**
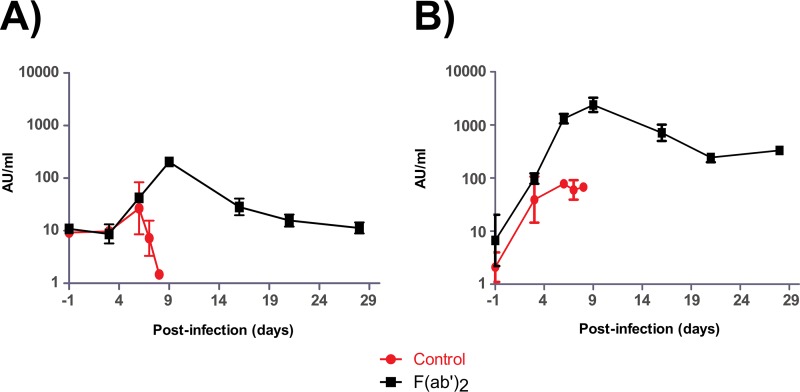
Humoral immunity of NHPs after EBOV challenge at 3 dpi. NHPs were given equine F(ab′)_2_ starting at 3 dpi. The amounts of circulating serum antibodies against EBOV were measured by ELISA at the times indicated and expressed as arbitrary units (AU) per milliliter of serum. (A) IgM. (B) IgG.

Serum biochemistry results showed no substantial changes in the activities or concentrations of alanine aminotransferase (ALT), alkaline phosphatase (ALP), amylase (AMY), total bilirubin (TBIL), blood urea nitrogen (BUN), or glucose (GLU) in the F(ab′)_2_-treated NHPs (Fig. 3G to L). In contrast, control animals showed increased ALT, ALP, TBIL, BUN, and GLU levels, as well as decreased AMY levels, which are markers of organ damage and are known to fluctuate with EVD progression. Viremia, as well as shedding via the nasal, oral, and rectal mucosa, was detected by real-time quantitative PCR (RT-qPCR) in both control NHPs (Fig. 4A to D). In contrast, transient viremia and shedding via the oral route were detected for 1 of 4 F(ab′)_2_-treated NHPs. When these data were taken together, F(ab′)_2_ appeared to be effective at postexposure treatment of infected NHPs, and the animals did not become severely ill. Surviving F(ab′)_2_-treated animals had detectable levels of circulating serum IgM and IgG after challenge, which were not observed in phosphate-buffered saline (PBS)-treated control animals (Fig. 5A and B).

### Efficacy of F(ab′)_2_ at 5 dpi against EBOV in NHPs.

**FIG 6:**
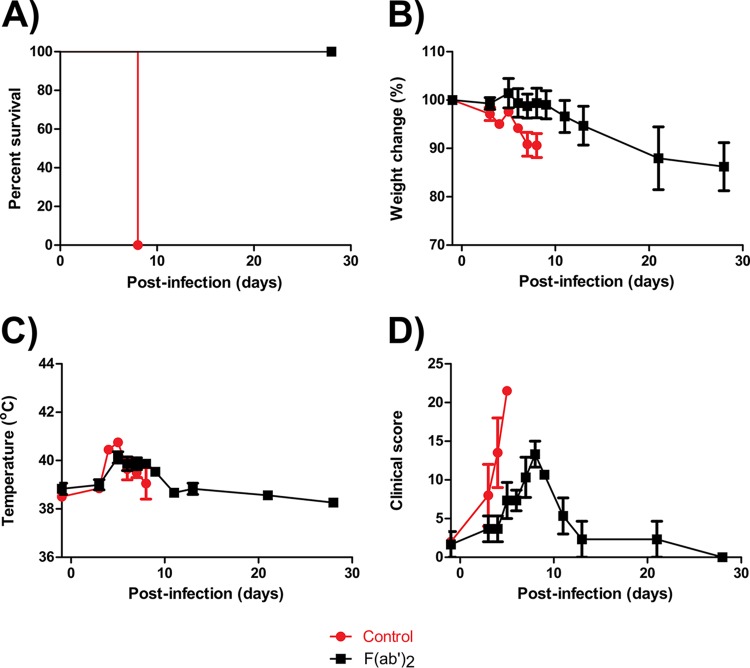
Survival rates and clinical findings for NHPs after EBOV challenge at 5 dpi. NHPs were given equine F(ab′)_2_ starting at 5 dpi. (A) Survival rates. (B) Percent weight changes. (C) Body temperatures. (D) Clinical scores.

Results showed that all F(ab′)_2_-treated animals survived challenge (Fig. 6A). A loss of body weight was observed for 1 of 3 F(ab′)_2_-treated NHPs (Fig. 6B). Fever was observed for all F(ab′)_2_-treated animals at 5 to 11 dpi but resolved afterwards (Fig. 6C). All F(ab′)_2_-treated NHPs were observed to be symptomatic at the beginning of treatment, and symptoms became progressively worse until 8 dpi. The animals fully recovered by 28 dpi, and no signs of disease were observed (Fig. 6D). In contrast, control animals succumbed to infection by 8 dpi, with pronounced weight loss, fever, and increases in clinical scores to >20.

**FIG 7:**
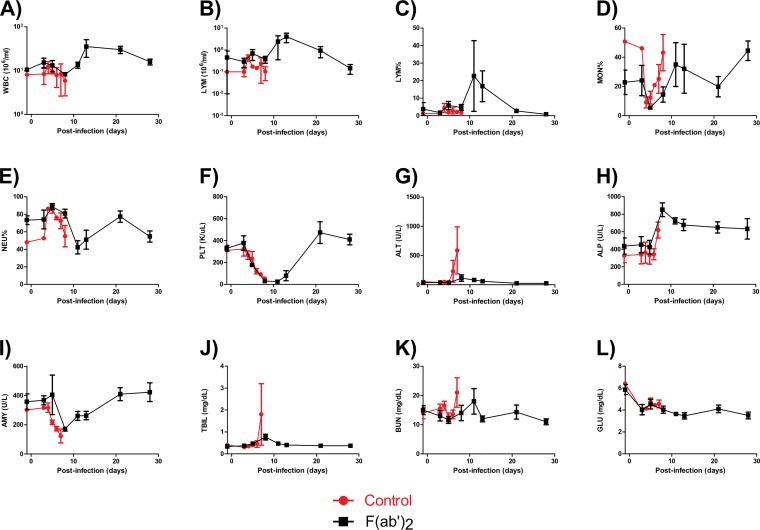
Hematology and serum biochemistry findings for NHPs after EBOV challenge at 5 dpi. NHPs were given equine F(ab′)_2_ starting at 5 dpi. (A) WBC counts. (B) LYM counts. (C) LYM percentages. (D) MON percentages. (E) NEU percentages. (F) PLT counts. (G) ALT levels. (H) ALP levels. (I) AMY levels. (J) TBIL levels. (K) BUN levels. (L) GLU levels.

**FIG 8:**
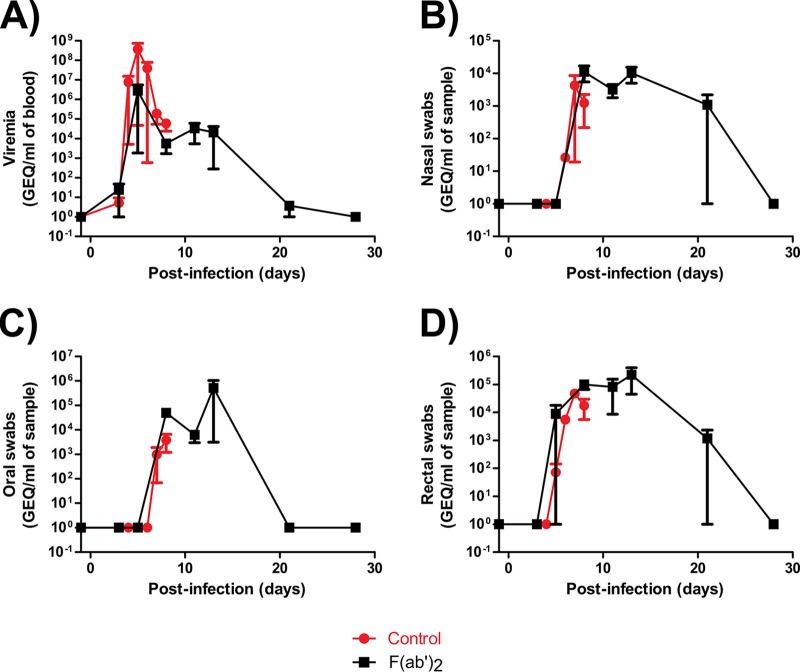
Viremia and shedding of NHPs after EBOV challenge at 5 dpi. NHPs were given equine F(ab′)_2_ starting at 5 dpi. (A) Viremia. (B) Nasal swabs. (C) Oral swabs. (D) Rectal swabs.

**FIG 9:**
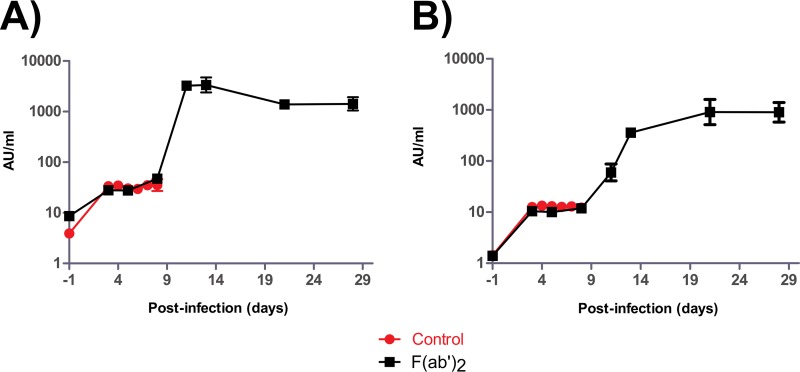
Humoral immunity of NHPs after EBOV challenge at 5 dpi. NHPs were given equine F(ab′)_2_ starting at 5 dpi. The amounts of circulating serum antibodies against EBOV were measured by ELISA at the times indicated and expressed as arbitrary units (AU) per milliliter of serum. (A) IgM. (B) IgG.

Complete blood count results showed that, by the start of treatment at 5 dpi, all F(ab′)_2_-treated animals had decreases in WBC counts, LYM counts, and LYM percentages, fluctuating MON percentages and NEU percentages, and substantial decreases in PLT counts (Fig. 7A to F), consistent with EVD. However, increases in all of the aforementioned markers were subsequently observed, suggesting that F(ab′)_2_ treatment provided the host sufficient time to generate its own immune responses. Control animals showed largely the same trends for these markers but did not recover from the infection. Serum biochemistry results showed that all F(ab′)_2_-treated animals had increased ALT, ALP, TBIL, and BUN levels and decreased AMY and GLU levels during the course of the experiment (Fig. 7G to L), suggesting that the animals had suffered organ damage. Control animals again showed the same trends, but the greater magnitude of marker changes indicated that these NHPs had more severe EVD. Analysis of viremia and virus shedding by RT-qPCR showed that the animals had, on average, over 10^6^ EBOV genome equivalents (GEQ) per milliliter of blood by the start of F(ab′)_2_ treatment, but values decreased to undetectable levels by 28 dpi (Fig. 8A). Virus shedding via the nasal and oral routes was not observed until 8 dpi and reached 10^5^ to 10^6^ GEQ/ml of sample but resolved by 28 dpi (Fig. 8B and C). Shedding via the rectal route was observed at 5 dpi and reached 10^4^ GEQ/ml of sample but resolved by 28 dpi (Fig. 8D). Surviving F(ab′)_2_-treated animals had detectable levels of circulating serum IgM and IgG after challenge, which were not observed in PBS-treated control animals (Fig. 9A and B).

## DISCUSSION

In this study, we successfully rescued 100% of EBOV-infected NHPs using an equine F(ab′)_2_ treatment initiated as late as 5 dpi, or 48 to 72 h before death. Passive immunotherapy against EBOV was investigated previously, with mixed results. During the 1995 EVD outbreak in Kikwit, Democratic Republic of the Congo, whole blood from survivors was passively transferred to ill patients, resulting in 7 of 8 survivors (22). However, a clinical trial in which 99 patients received two consecutive transfusions of 200 to 250 ml of convalescent plasma was not associated with a statistically significant improvement in survival rates (23). It should be noted, however, that levels of EBOV-specific neutralizing antibodies in the convalescent plasma were not tested; therefore, the quality of the plasma was neither standardized nor known.

Scientists also previously investigated the use of hyperimmune equine antisera against EBOV. This treatment (1 mg/kg) was shown to be 100% effective in baboons treated before challenge and 67% effective postexposure, but the challenge dose was low (10 to 30 PFU) (24, 25). Delays to viremia and the onset of clinical symptoms were observed, but the treatment (approximately 10 mg/kg) did not confer a survival benefit in cynomolgus macaques (26). However, passive immunotherapy with purified IgG (80 mg/kg) from NHPs that survived EVD was successful in conferring postexposure protection when treatment was initiated as late as 2 dpi (27), suggesting that higher doses of antisera are needed. Building on this, our study shows that viral neutralization via F(ab′)_2_ is sufficient to control EVD and is effective even when animals are symptomatic. However, it is known that immunoglobulin fragments usually have shorter half-lives than IgG and therefore must be administered frequently, as shown in the current study with seven injections. To develop this product further, it will be important to assess whether the half-life of F(ab′)_2_ can be extended using strategies such as protein conjugation (28) in order to confer complete protection with fewer treatment doses, lower F(ab′)_2_ concentrations, and reduced infusion times, as work conditions in EBOV treatment centers in Africa are demanding and multiple infusions can further complicate the work of primary caregivers in the field.

While the results suggested that F(ab′)_2_ slowed EBOV infection in NHPs enough that the animals had sufficient time to develop a specific protective response, it is not known whether the animals would survive a rechallenge with EBOV, and this needs to be investigated in the future. A previous study showing complete protection of ZMAb-treated NHPs against an EBOV rechallenge 10 weeks after the initial infection suggested that surviving animals might have developed protective immunity. Without directly comparing different treatments under the same challenge conditions, we cannot conclude whether F(ab′)_2_ is superior or inferior to ZMapp or other antibody-based products. However, our results convincingly show that F(ab′)_2_ is effective as a postexposure treatment (as late as 5 dpi) in NHPs and should be included in clinical trials as soon as possible. Due to well-established production protocols and a good safety record, F(ab′)_2_ should be considered as an alternative to MAbs for large-scale treatment of patients during EBOV epidemics.

## MATERIALS AND METHODS

### Ethics statement.

The experiments were performed in the biosafety level 4 (BSL4) laboratory at the National Microbiology Laboratory in Winnipeg, Canada, under Animal Use Document (AUD) H-16-015. The experiments described in the AUD were approved by the Animal Care Committee (ACC) at the Canadian Science Center for Human and Animal Health and adhered to the national regulations and guidelines outlined by the Canadian Council on Animal Care. To ameliorate suffering, animals were euthanized with an intracardiac injection of pentobarbital sodium if the clinical score reached ≥25 (or ≥20 for control NHPs), following guidelines set by the ACC.

### *In vitro* and *in vivo* study design.

Neutralization assays were performed *in vitro* using a previously published protocol (10). Equine F(ab′)_2_ was generated as described in a previous study (17), and the same batch was used in this study. In the pilot NHP experiment, rhesus macaques were infected with 1,000 times the 50% tissue culture infectious dose (TCID_50_) of EBOV-Makona-C07. Beginning at 3 dpi, animals (*n* = 4) were treated with 100 mg/kg of F(ab′)_2_ once daily until 7 dpi (5 doses), with identical doses also being given at 9 and 11 dpi. A control animal (*n* = 1) was given identical volumes of PBS in place of F(ab′)_2_. Another control animal (*n* = 1) was left untreated. Survival rates, in addition to changes in clinical, hematological, biochemical, and virological parameters, were monitored for 28 dpi using previously published protocols (10).

To test whether F(ab′)_2_ was effective later in the course of EVD (when the animals were symptomatic), naive rhesus macaques were first infected with 1,000 TCID_50_ of EBOV-Makona-C07. Beginning at 5 dpi, animals (*n* = 3) were treated with 100 mg/kg of F(ab′)_2_ once daily until 9 dpi (5 doses), with identical doses also being given at 11 and 13 dpi. Control animals (*n* = 2) was given identical volumes of PBS in place of F(ab′)_2_. All parameters were monitored for 28 dpi in a manner similar to that of the pilot experiment.

### Enzyme-linked immunosorbent assay.

Enzyme-linked immunosorbent assays (ELISAs) were performed with sera harvested from NHPs, as described previously (29).
